# A Study on Causes and Types of Abnormal Increase in Infants’ Head Circumference in Kashan/Iran

**Published:** 2013

**Authors:** Ahmad TALEBIAN, Babak SOLTANI, Alireza MORAVVEJI, Ladan SALAMATI, Majid DAVAMI

**Affiliations:** 1 Department of Pediatrics, Kashan University of Medical Sciences, Kashan, Iran.; 2 Department of Community Medicine, Trauma Research Center, Kashan University of Medical Sciences, Kashan, Iran.; 3 Department of Dermatology, Kashan University of Medical Sciences, Kashan, Iran.

**Keywords:** Macrocephaly, Infants, Hydrocephalus, Fontanel

## Abstract

**Objective:**

Head circumference is a valuable index of brain growth and its disturbances can indicate different disorders of nervous system. Abnormal increased head circumference (macrocephaly) is common and observed in about 2% of infants. In this study, the causes and clinical types of abnormal increase in infants’ head circumference were investigated in Kashan, Iran.

**Materials and Methods:**

This cross-sectional study was performed on 90 infants less than 2 years of age with abnormal increase in head circumference in Kashan, during 2009-

2011. The data were collected by history taking, physical examination, growth chart, and imaging.

**Results:**

65 (72%) cases out of 90 infants were male and 25 ( 28%) cases were female. Fifty-three (58.8%) cases had familial megalencephaly, 30 (33.4%) had hydrocephalus, and other causes were observed in 7 (7.8%) cases. Eighty-three percent of Infants with familial megalencephaly and

50% with hydrocephalus had normal fontanels. In 90.6% of cases with familial megalencephaly, family history for large head was positive. Motor development was normal in 100% of cases with familial megalencephaly and 76.7% of hydrocephalic infants.

**Conclusion:**

Familial megalencephaly was the most common cause of macrocephaly in the studied infants, and most of them had normal physical examination and development, so, parental head circumferences should be considered in the interpretation of infant’s head circumference and in cases of abnormal physical examination or development, other diagnostic modalities, including brain imaging should be done.

## Introduction

Measurement of head circumference (HC) is a valuable index for monitoring brain growth, especially when done consecutively and recorded in a growth chart. It can be influenced by genetic and environmental factors (1-3). Abnormal HC can be a sign of various nervous system disorders (4-5). Macrocephaly is defined as HC greater than two standard deviations (SDs) above the mean for age, sex, and gestation. HC is common and seen in 2% of infants (6-7) and could be the first sign of genetic and acquired brain disorders (4,6,8). Macrocephaly can be caused by increased brain size (megalencephaly) or cerebrospinal fluid (hydrocephalus). Macrocephaly is usually a genetic- based disorder, but sometimes an important condition, such as hydrocephalus may be found and early diagnosis and intervention are helpful (6,9,10). For differentiation of causes of macrocephaly, imaging modalities, such as sonography, computed tomography (CT) scan and Magnetic Resonance Imaging (MRI) are used (11-13). Familial megalencephaly is the most common cause of macrocephaly with large head at birth, typically 2-4 cm above the 90th percentile, but parallel to the 98th percentile without any clinical manifestations and positive family history of large head (14). The second common cause is external hydrocephalus, which characterized by accumulation of cerebrospinal fluid in the subarachnoid space of frontal or frontoparietal areas with a prominent interhemispheric fissure and normal ventricles (15). As macrocephaly is a common disorder and many cases are referred from health- care centers, therefore, this study was done with the aim of evaluating the causes and prognostic factors of macrocephaly in infants.

## Materials and Methods

This cross-sectional survey was done on 90 infants with abnormal increase in HC referred to the Pediatric Neurologic Clinic of Shahid Beheshti Hospital, between January 2009 and January 2011. An exact physical and neurological examination was performed by a pediatric neurologist and the infants’ HC was checked and plotted on a standard growth chart (NCHS) by him (16). macrocephaly is diagnosed, when HC is greater than 2 standard deviations above the mean for age and sex (4). A complete history was obtained from the parents about motor and cognitive development of their infants and the growth charts were evaluated from birth regarding the onset age and quality of macrocephaly. If the macrocephalic infants had normal motor-cognitive and physical examinations, brain sonography was done and if there was abnormality in sonography, brain MRI was implemented. In cases with abnormal motor-cognitive or physical examination, brain MRI was done at first. According to the history, physical and neurological examinations, and neuroimaging, the patients were divided into 3 groups: familial megalencephaly, hydrocephalus, and miscellaneous. Sample size was estimated according to CI=95%, prevalence of macrocephalus of 5%, and d=5% (17). The infants’ data were collected in questionnaires and the statistical analysis was done by descriptive statistics and chi-square test using SPSS-13 software.

## Results

Ninety infants with macrocephaly were evaluated during two years. Sixty-five (72%) infants were male and 25 (28%) were female. Fifty-three (58.8%) had familial megalencephaly, 30 (33.4%) had hydrocephalus, and 7 (7.8%) had miscellaneous causes of macrocephaly, including intracranial hemorrhage (3 cases), sagittal synostosis (2 cases), achondroplasia (1 case), and glutaric aciduria type 1. The mean ages of the onset of abnormal increase of HC in familial megalencephaly, hydrocephalus, and miscellaneous were 2 months, 3 months, and 1.6 months old, respectively, and the mean age of them was 8.64 months.

The birth HC≥97 percentile in familial megalencephaly, hydrocephalus, and miscellaneous causes were 49.1%, 43.4%, and 57.1%, respectively (Table-1). Eighty percent of familial megalencephalic infants had normal fontanel, but 50% and 57.1% of cases, respectively, had normal frontal in hydrocephalus cases and miscellaneous cases, so the relation between fontanel condition and causes of increased HC was statistically significant (p=0.003) (Table-2). Normal sutures were 100% in familial megalencephalic cases, while they were 80% in hydrocephalus and miscellaneous cases, therefore, there was a statistically significant relation between suture condition and causes of increased HC (p=0.004). There was no statistically significant relationship between causes of increase HC and sex (p=0.359). All cases of familial megalencephaly, 76.7% of hydrocephalic infants, and 71.4% of miscellaneous groups had normal motor development (Table-3). In hydrocephalic patients, 93.3% of external hydrocephalus, 60% of communicating hydrocephalus, and 50% of obstructive hydrocephalic cases had normal development. In familial megalencephalic infants, 90.6% of cases had positive family history for large head (43.4% of them in father, 26.4% in mother, and the remainder in siblings). It was 48.3% and 57.2% in hydrocephalic and miscellaneous cases, respectively. Sudden onset of abnormal increase in HC occurred in 3.9% of familial megalencephaly, 26.7% of hydrocephalic, and 50% ofmiscellaneous cases, respectively. Out of 30 infants with hydrocephalus, 50% had obstructive or communicating hydrocephalus and 50% had external hydrocephalus (benign increase in subarachnoid space).

## Discussion

In this study, 72% out of 90 infants were male and 28% were female. In Medina et al.’s investigation in 2001, 67% of macrocephalic infants were male and 33% were female (17). Day et al. showed familial megalencephaly and external hydrocephalus as the most common causes of macrocephaly and there were more prevalent in males (14). In Lorber et al.’s study the frequency of macrocephaly in boys was four-fold higher than girls (18) and these results are compatible with our findings. In their research, 58.8% of infants had familial megalencephaly. Day et al. showed that familial megalencephaly is the most common cause of macrocephaly (14) and these infants had large head at birth with rapid growth rate during first few months of life, as HC was above 98 percentile (14). On the other hand, in a study by Lorber et al, 20% of macrocephalic infants had familial megalencephaly (18) and this difference may be due to genetic varietion in different geographic areas. In this study, 33.4% of infants had hydrocephalus and 50% had external hydrocephalus. Zahl et al. studied on 298 admitted children with macrocephaly, and 173 of their cases had hydrocephalus (19). The reason of this discrepancy may be because the study of Zahl et al. was done on hospitalized cases and our study was conducted on outpatients. In this study, the mean onset age of increase in HC was 3 months old and the mean age was 9.1 months old, while in Zahl et al’s investigation, they were 4.8 and 8.7 months of age, respectively (19). In the present study, 90.6% of familial megalencephaly had a positive family history of macrocephaly, that in 43.4% of them, their fathers had large head. In Lorber et al’s study, 50% of familial megalencephalic infants had positive family history of large head (18). In Day’s study, 10 out of 15 infants with familial megalencephaly had a history of increased HC in their fathers (14). In our investigation, 57.1% of external hydrocephalic cases had a positive family history of large head, while in Alvarez et al.’s and Yew et al.’s studies, they were 88% (20) and 10% (21), respectively. In this study, 83% of infants with familial megalencephaly had normal fontanel and 100% had normal motor and cognitive development, whereas in Lorber’s study 6.4% of their cases had delayed motor and cognitive development (18). In our study, 50% of hydrocephalic cases had normal fontanel, while it was 83% in familial megalencephaly, it can show that a relationship exists between fontanel examination and the cause of macrocephaly, therefore, neuroimaging is imperative in cases with abnormal fontanels. In our study, 76.7% of hydrocephalic cases had normal motor development, it was 93.3% in external hydrocephalus, 50% in obstructive, and 60% in communicating hydrocephalus. In Alvarez et al,’s study, from 36 infants with external hydrocephalus, three cases had mild developmental delay and in one case, it was severely impaired (20). In Yew et al.’s investigation, 21% of 99 infants with external hydrocephalus had developmental delay (21). In Muenchberger et al.’s study, 2 out of 15 cases with external hydrocephalus had delayed motor development and 2 cases had delayed speech development (22). In our research, 5 out of 30 hydrocephalic infants required shunt placement (1 case of communicating hydrocephalus and 4 cases of obstructive hydrocephalus) and no cases of external hydrocephalus needed shunt. No cases of external hydrocephalus required shunt placement in Alvarez et al’s and Yew et al’s studies (20,21).

In Conclusion, in this study, the most common cause of macrocephaly was familial megalencephaly and the second common cause was external hydrocephalus. In most of these infants, neurologic examination, fontanels, and development were normal. An exact history, and neurological and physical examinations are mandatory in cases of abnormal increase in HC, and if any abnormality is found, diagnostic modalities, including brain imaging is prudent.

**Table 1:** Frequency of Increased Head Circumference Causes According to Birth Head Circumference

**Macrocephaly causes**	**Familial megalencephaly No.** **(percent)**	**Hydrocephalus** **No. (percent)**	**Other causes No. (percent)**	**Total No. (percent)**
**Birth HC percentile**				
≤25	5 (9.4%)	5 (16.6%)	2 (28.6%)	12 (13.3%)
50	8 (15.0%)	6 (20%)	1 (14.3%)	15 (16.7%)
75	13 (24.6%)	6 (20%)	0 (0%)	19 (21.1%)
95	1 (1.9%)	0 (0%)	0 (0%)	1 (1.1%)
≥97	26 (49.1%)	13 (43.4%)	4 (57.1%)	43 (47.8%)
Total	53 (100%)	30 (100%)	7 (100%)	90 (100%)

**Table 2:** Frequency of Increased Head Circumference Causes According to Fontanel Condition

**Macrocephaly Causes**	Familial Megalencephaly No. (percent)	Hydrocephalus No. (percent)	Other Causes No. (percent)	Total No. (percent)	Statistical Comparison
**Fontanel condition**					
Normal	44 (83.0%)	15 (50.0%)	4 (57.1%)	63 (70.0%)	p=0.003
Abnormal	9 (17.0%)	15 (50.0%)	3 (42.9%)	27 (30%)	
Total	53 (100%)	30 (100%)	7 (100%)	90 (100%)	

**Table 3:** Frequency of Increased Head Circumference Causes According to Motor Development

Macrocephaly causes	Familial megalencephaly No. (percent)	Hydrocephalus No. (percent)	Other causes No. (percent)	Total No. (percent)	Statistical comparison
Motor development					
Normal	53 (100%)	23 (76.7%)	5 (71.4%)	81 (90%)	p=0.001
Abnormal	0 (0%)	7 (23.3%)	2 (28.6%)	9 (10%)	
Total	53 (100%)	30 (100%)	7 (100%)	90 (100%)	
